# Pediatric omental torsion: A rare radiologic diagnostic challenge

**DOI:** 10.1016/j.radcr.2026.03.065

**Published:** 2026-05-07

**Authors:** Burak Öztürk, Ayşe Aysel Köseoğulları Bulut

**Affiliations:** a Department of Radiology, Ünye State Hospital, Ordu, Türkiye; b Department of Pediatric Surgery, Fatsa State Hospital, Ordu, Türkiye

**Keywords:** Primary omental torsion, Acute abdomen, Pediatrics, CT whirlpool sign, Conservative management, Laparoscopic omentectomy

## Abstract

Omental torsion is a rare cause of acute abdomen, with approximately 400 cases reported in the literature, only a few of which were diagnosed preoperatively. The number of reported cases in pediatric patients is relatively limited. Diagnosis is challenging because the nonspecific clinical presentation often mimics other acute abdominal conditions, particularly appendicitis. We report the case of a 15-year-old male who was presented to the emergency department with a 5-day history of abdominal pain. Clinical and laboratory findings suggested an inflammatory process; however, imaging studies demonstrated a normal appendix. Computed tomography revealed the *vascular pedicle sign* and the *whirlpool sign*, leading to a preliminary diagnosis of omental torsion. The patient underwent successful laparotomic omentectomy due to persistent severe pain and suspected recurrence despite conservative management. They were discharged on postoperative day 3. Omental torsion should be included in the differential diagnosis of patients presenting with acute abdominal pain. This case highlights the importance of radiological evaluation in pediatric patients, as accurate imaging can inform appropriate clinical management and help avoid unnecessary surgery. Nevertheless, laparoscopic surgery remains a valuable option when conservative management proves ineffective.

## Introduction

Omental torsion is a rare cause of an acute abdomen. The incidence reported in the literature varies widely, ranging from 0.0016% to 0.37% [1,2]. This wide variation reflects differences in study populations, diagnostic criteria, and reporting methods across the published literature.

While around 400 cases of omental infarction have been reported to date, its true incidence remains unclear [3]. Pediatric cases are even rarer, accounting for around 15% of all reported cases. According to previous reports, the estimated incidence of omental infarction in children undergoing laparotomy for suspected appendicitis ranges from 0.024% to 0.1% [4,5]. The relatively underdeveloped omentum in children has been suggested as a factor that contributes to a lower risk of torsion [5].

The hallmark pathological feature is the rotation of the omentum around its longitudinal axis, leading to venous congestion followed by arterial ischemia, ultimately resulting in focal hemorrhage and fat necrosis [1,6,7]. Accurate diagnosis is challenging due to nonspecific clinical findings [6]. Clinical symptoms and signs often resemble those of other causes of abdominal pain, particularly acute appendicitis [1,4].

Preoperative diagnosis relies on the characteristic appearance of omental torsion on computed tomography (CT) scans [1,4,8]. Depending on clinical presentation and diagnostic certainty, management consists of either conservative treatment or surgical resection of the affected omentum [1,4,6,[8], [9], [10]]. In this report, we present a case of omental torsion that presented to our clinic with an acute abdomen. The diagnosis was supported by characteristic rotational patterns on CT scans and confirmed through surgical exploration and pathological examination.

## Case report

A 15-year-old male patient was presented to the emergency department with a 5-day history of abdominal pain. Initially located in the umbilical region, the pain gradually increased in severity and eventually became localized to the lower abdominal quadrants. He denied experiencing nausea or vomiting. For the past 2 years, he had experienced similar episodes of abdominal pain in the umbilical region approximately every six months, with each episode lasting up to one week. Previous medical evaluations had attributed his symptoms to infectious causes, and he had received appropriate medical treatment. The patient reported intermittent episodes of diarrhea, but had no significant history of constipation except during infancy. Notably, he underwent right inguinal hernia repair surgery at the age of 2.

A physical examination revealed stable vital signs and a body mass index of 21. Abdominal examination revealed increased tenderness, guarding, and rebound tenderness, particularly in the right upper and lower quadrants. Examination of other systems was unremarkable.

**Fig. 1:**
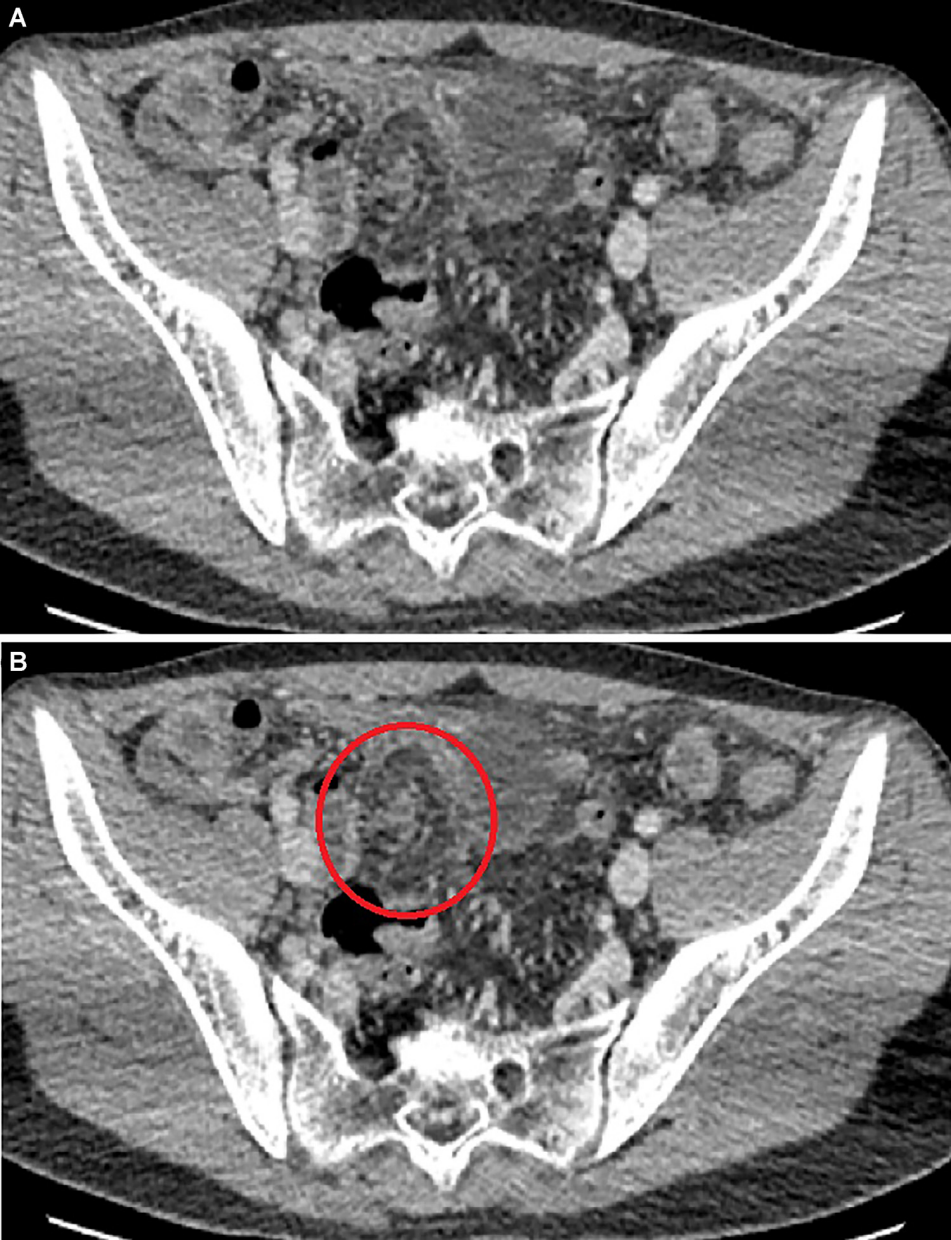
Axial contrast-enhanced CT view showing omental torsion. **(A)** The whirlpool sign is highlighted by the red circle **(B).**

**Fig. 2:**
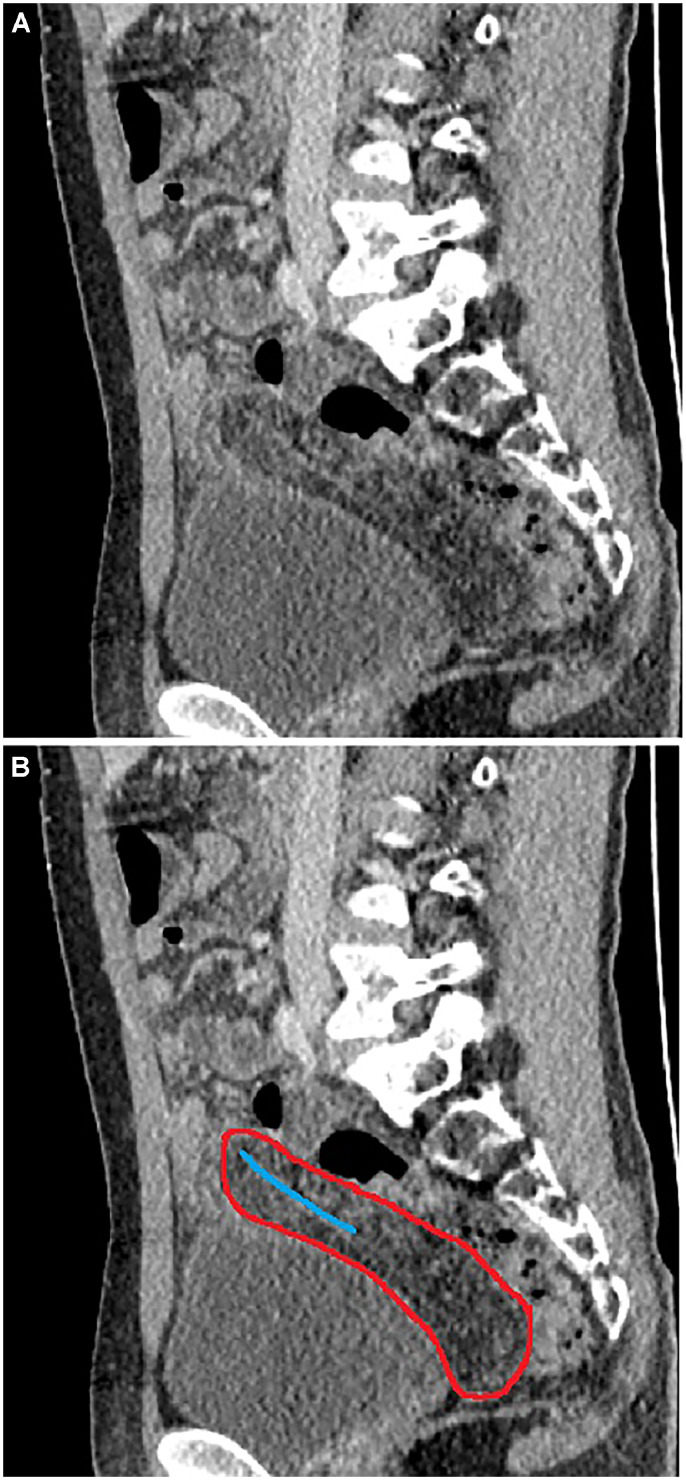
Sagittal contrast-enhanced CT image of volvulus. **(A)** The red-marked area shows the omental volvulus extending into the Douglas pouch, the blue line indicates the vascular pedicle sign **(B).**

Laboratory tests revealed leukocytosis (15,000/mm³) and elevated C-reactive protein (150 mg/L), with no other notable findings. An abdominal ultrasound was performed using a Toshiba Aplio 500 Ultrasound with convex (3.5 MHz) and linear (10–14 MHz) probes. The ultrasound demonstrated inflamed fatty tissue extending craniocaudally towards the rectovesical space, accompanied by incomplete twisting at the proximal segment and a small amount of free fluid in the right lower quadrant. The differential diagnosis included Meckel's diverticulum and omental torsion. Imaging findings were unavailable for review and could therefore not be presented. Thin-section (1 mm), contrast-enhanced abdominal computed tomography (Toshiba Alexion 16, Japan) revealed a normally positioned, non-inflamed appendix. However, the omentum was observed to be twisted around itself, extending posteriorly to the Douglas pouch with the characteristic vascular pedicle and whirlpool signs (Figs. 1 and 2). The CT examination was performed using a low-dose protocol (120 kVp, 50 mAs), with total collimation of 16 × 1.0 mm and a pitch of 0.938. Images were reconstructed at a slice thickness of 1 mm using a soft tissue kernel. The CTDIvol was 4.5 mGy, consistent with ALARA (As Low As Reasonably Achievable) principles.

**Fig. 3:**
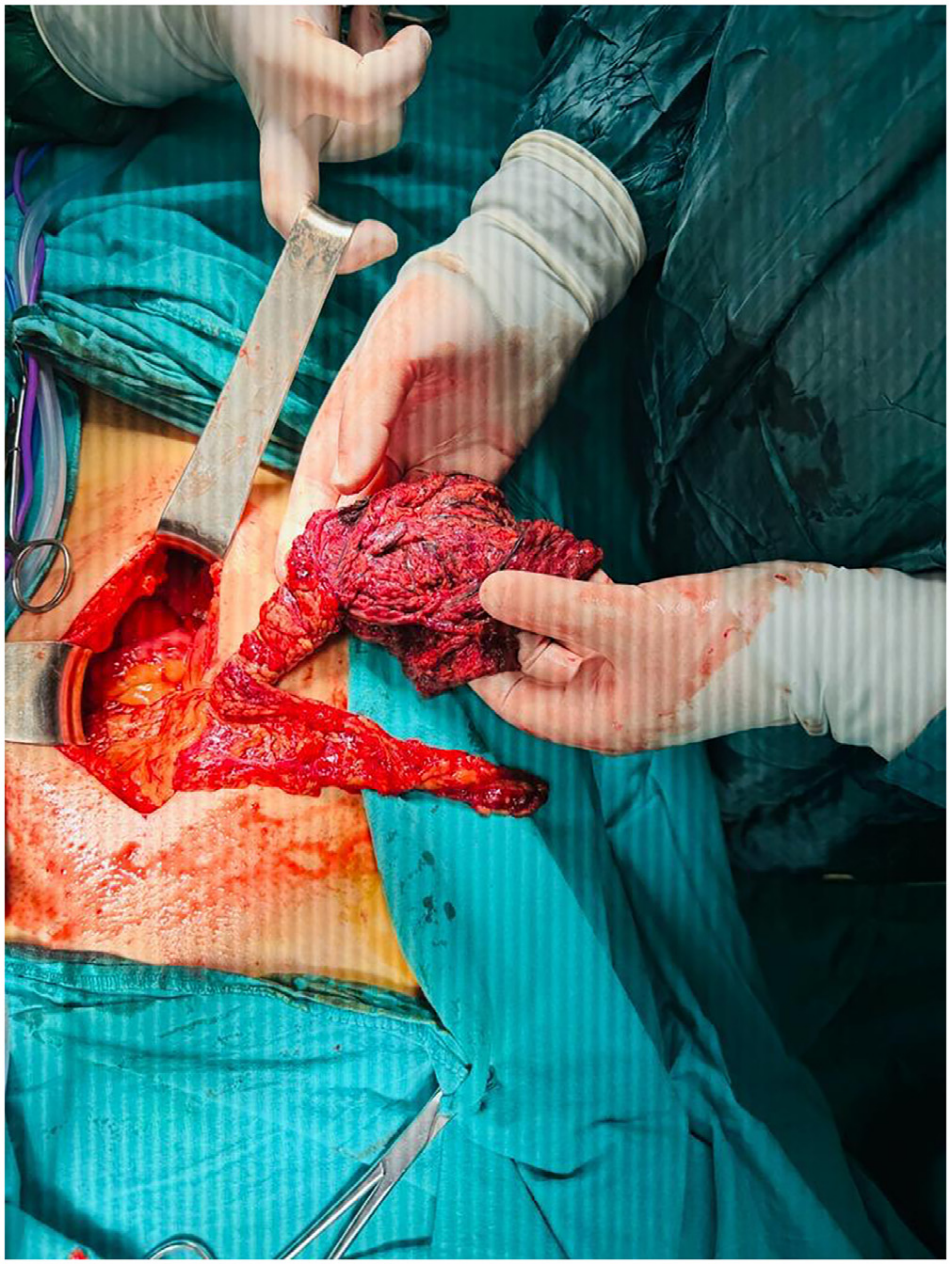
Twisted omental segment with distal necrosis in the patient undergoing laparotomy.

Despite the preoperative CT diagnosis of omental torsion, an exploratory laparotomy via a midline incision was performed due to the severity of the clinical symptoms and established institutional protocols for an acute abdomen. During the procedure, serohemorrhagic fluid was found in the Douglas pouch. The appendix was found to be in its normal anatomical position and appearance. The omentum extended towards the right lower quadrant and was adhered to the lower portion of the ileocecal valve. The omentum was twisted 3 times around its mid-portion axis. The distal part of the torsion appeared completely necrotic and fragile (Fig. 3). The affected segment was resected and a partial omentectomy was performed. An appendectomy was also carried out.

Postoperatively, the patient was treated with parenteral antibiotics, including a cephalosporin and metronidazole. Clinical recovery was uneventful, and he was discharged in good condition on the third postoperative day. Pathological examination of the resected omentum confirmed fat necrosis and hemorrhagic foci, along with serositis.

## Discussion

The omentum is a 4-layered fold of peritoneum that extends from the stomach to the transverse colon [2,4]. It is divided into a greater omentum and a lesser omentum.

Greater omental torsion is a rare clinical condition characterized by the omentum twisting around a fixed point, typically in a clockwise direction. This leads to ischemia and acute abdominal pain [6,8]. Despite being a well-documented cause of abdominal pain, its rarity often leads to it being overlooked in the differential diagnosis of an acute abdomen [2]. Fewer than 400 cases have been reported [3]. The incidence is higher in males and individuals younger than 50 years, with male-to-female ratios ranging from 2:1 to 5:1 [1,7,10]. According to previous reports, the estimated incidence of omental infarction in children undergoing laparotomy for suspected appendicitis ranges from 0.024% to 0.1% [4,5].

Venous return is compromised during torsion, leading to congestion and edema of the distal omentum. If arterial occlusion ensues, hemorrhagic infarction and fat necrosis occur [1,6,11].

Omental torsion is classified as primary or secondary. Primary omental torsion accounts for approximately one-third of all omental torsion cases [2]. Primary omental torsion is unipolar, occurring without an identifiable secondary cause, whereas secondary torsion is bipolar and typically associated with fixation of both the distal and proximal omentum due to adhesions, hernias, postoperative scarring, or intra-abdominal inflammation [1,2,8,9,11]. Predisposing factors for primary torsion include anatomical variants such as tongue-shaped projections, accessory omentum, or a narrow pedicle, in addition to venous redundancy [1,2,9,11]. Primary torsion generally affects obese children older than 4 years [1,2,4,8].

Despite differing etiologies, both forms share common risk factors: trauma, obesity, intraabdominal anomalies, hyperperistalsis, and overeating [1,2,4].

In this reported case, despite the patient having a history of right inguinal hernia repair, no abnormalities were found in the inguinal canal orifice or the previous surgical site.

The clinical manifestations are non-specific and depend on the degree and duration of torsion [9]. The defining clinical presentation is the sudden onset of persistent, non-radiating abdominal pain, which is usually constant and gradually becomes more intense. The anatomical region most commonly affected is the right lower quadrant. However, the location of the pain can vary depending on the quadrant in which the torsion occurs. Due to preserved bowel peristalsis, gastrointestinal symptoms such as nausea and vomiting are absent in more than half of patients [1,2,4,6,8,9]. Low-grade fever and a palpable abdominal mass may be present, and recurrent symptoms may suggest intermittent torsion, as seen in this patient’s previous non-specific abdominal pain episodes. Laboratory studies often reveal leukocytosis and elevated C-reactive protein levels [1,2,4,6,8,9].

The differential diagnosis of omental torsion is broad, including acute appendicitis, epiploic appendagitis, Meckel’s diverticulitis, acute cholecystitis, diverticulitis, volvulus, ovarian torsion, ectopic pregnancy, and salpingitis. CT can help narrow the differential diagnosis by demonstrating the absence of typical findings of acute appendicitis and diverticulitis, such as an inflamed appendix or diverticulum, significant bowel wall thickening, or paracolic abscess formation [11]. Fat-containing lesions such as lipoma, liposarcoma, teratoma, angiomyolipoma, mesenteric lipodystrophy, and pseudomyxoma peritonei may cause radiological confusion. However, diagnostic uncertainty is usually limited because of the different clinical presentations [1,2,4,11]. Omental infarction, a rare cause of right lower quadrant pain in pediatric patients, should also be considered in the differential diagnosis [12]. Omental infarction typically has a triangular configuration and usually involves the inferior aspect of the right side of the omentum. It is characteristically located between the anterior abdominal wall and the transverse or ascending colon, corresponding to the position of the greater omentum [12]. Epiploic appendagitis is typically located adjacent to the colonic wall and appears as a well-defined round or oval lesion. On CT, it presents as a fat-density lesion surrounded by a hyperattenuating inflammatory rim, whereas on ultrasound it appears as a hyperechoic, oval, noncompressible mass. Thickening of the adjacent colonic wall is usually absent [11,13].

High-resolution CT and ultrasonography have improved the accuracy of preoperative diagnosis [1,2,6]. If preoperative imaging confirms the diagnosis, conservative management may be attempted, since omental torsion is usually self-limiting and has a low incidence of complications [7,8]. Imaging features in pediatric patients are generally similar to those described in adults [12].

Ultrasound findings include an incompressible, avascular, hyperechoic mass that is adherent to the abdominal wall and surrounded by a hypoechoic halo [4,8]. As in the present case, when torsion is severe, the twisted pedicle sign may be evident. Imaging findings are typically detected at the site of maximal tenderness [13]. Contrast-enhanced ultrasound (CEUS) can provide further information by showing areas that do not enhance, which correspond to perfusion defects in the omental tissue. This can help to confirm the diagnosis of omental infarction [13,14].

Key signs such as the vascular pedicle sign and the whirlpool sign, which are indicative of twisted mesenteric vessels, can be revealed by CT imaging [1,4,8]. However, these signs may not be presented in all cases, particularly when the axis of rotation is not perpendicular to the imaging plane. CT scan findings in omental torsion include a dense fatty mass shaped like a cake or an oval with hyperattenuating streaks [4]. A single image is often insufficient for a diagnostic evaluation in computed tomography; rather, recognition and correct interpretation of the whirlpool sign demand the assessment of serial cross-sectional images. CT plays an important role in ruling out alternative diagnoses. However, radiation protection is particularly important in pediatric patients, and imaging protocols should strictly adhere to the ALARA principle. Ultrasonography should be the first-line imaging modality as it avoids ionizing radiation; CT should only be used in selected cases with inconclusive findings or suspected complications [15].

Treatment strategies are debated, with conservative management or surgical intervention employed depending on the clinical scenario. Conservative management has increasingly been reported as a safe and effective option in pediatric patients with uncomplicated omental torsion [16]. When conservative treatment fails or in cases of diagnostic uncertainty, laparoscopy is recommended for definitive management, with laparoscopic omentectomy being the treatment of choice [1,2,4,8,9,11].

## Conclusion

Omental torsion represents a rare and diagnostically challenging cause of acute abdominal pain in children. While the diagnosis is often made postoperatively, advances in imaging techniques allow for accurate preoperative diagnosis in most cases. Radiologic imaging, particularly computed tomography, plays a pivotal role in establishing the preoperative diagnosis through its characteristic features. This case highlights the importance of radiologic evaluation in pediatric patients, as accurate imaging can support appropriate clinical management and help avoid unnecessary surgery. However, when conservative treatment fails or in cases of diagnostic uncertainty, diagnostic laparoscopy and resection of the affected omental segment remain the definitive treatment approach.

## Data availability

No datasets were generated or analyzed.

## Patient consent

Written informed consent was obtained from the patient’s parent for the publication of any clinical information, images, or other relevant data included in this manuscript. The parent has been informed that all personal identifiers will be removed to ensure anonymity.

## References

[1] Carrillo L.M., De Jesús Marín-López J., Díaz-Barrera O., Olvera-Rodríguez J.A., Gutiérrez-Gutiérrez L.Y., Herrera-Gutiérrez J. (2023). Omental torsion; an unusual case of acute abdomen. Case report. Int J Surg Case Rep.

[2] Tartar T., Bakal Ü, Saraç M., Genç E., Kazez A. (2018). Çocuklarda nadir bir akut batın nedeni: primer omentum torsiyonu. Tcdd.

[3] Park T.U., Oh J.H., Chang I.T., Lee S.J., Kim S.E., Kim C.W. (2012). Omental infarction: case series and review of the literature. J Emerg Med.

[4] Khalili E., Marashi M., Safarpanah M., Majidi S., Hesarooeyeh Z.G. (2022). Omental torsion mimicking acute appendicitis in a 7-year-old boy: a case report. J Med Case Reports.

[5] Maekawa S., Kamiyama M., Fujita C., Takao D., Sumi K., Watanabe K. (2025). Omental torsion diagnosed and treated with single-incision laparoscopic surgery in 2 pediatric patients: a case report. Surg Case Rep.

[6] Alexiou K., Ioannidis A., Drikos I., Sikalias N., Economou N. (2015). Torsion of the greater omentum: two case reports. J Med Case Rep.

[7] Saad E., Awadelkarim A., Agab M., Babkir A., Yeddi A. (2022). Omental fat torsion: a rare mimicker of a common condition. J Investig Med High Impact Case Rep.

[8] Dias S.J.T., Gobishangar S., Sureska G.M., Vaishnavi T., Priyatharsan K., Theepan J.M.M. (2023). Omental torsion - a mimicker of the acute appendicitis - a case report. Int J Surg Case Rep.

[9] Borgaonkar V., Deshpande S., Rathod M., Khan I. (2013). Primary omental torsion is a diagnostic challenge in acute abdomen—a case report and literature review. Indian J Surg.

[10] Tsironis A., Zikos N., Bali C., Pappas-Gogos G., Koulas S., Katsamakis N. (2013). Acute abdomen due to primary omental torsion: case report. J Emerg Med.

[11] Corvino A., Campanino M.R., De Rosa N., Corvino F., Gisonni P. (2020). Left-sided omental infarction without torsion: report of a case with radiologicpathologic correlation. Egypt J Radiol Nucl Med.

[12] Grattan-Smith J.D., Blews D.E., Brand T. (2002). Omental infarction in pediatric patients: sonographic and CT findings. Am J Roentgenol.

[13] Trovato P., Simonetti I., Verde F., Lomoro P., Vinci G., Tarotto L. (2020). Acute epiploic appendagitis: ultrasound and computed tomography findings of a rare case of acute abdominal pain and the role of other imaging techniques. Pol J Radiol.

[14] Safai Zadeh E., Görg C., Kuttkat P., Dietrich C.F., Westhoff C.C., Rodepeter F. (2022). The value of contrast-enhanced ultrasound (CEUS) in the detection of perfusion disturbances in abdominal wall hernias compared with surgical and histological assessment. Diagnostics (Basel).

[15] Zacharias C., Alessio A.M., Otto R.K., Iyer R.S., Philips G.S., Swanson J.O. (2013). Pediatric CT: strategies to lower radiation dose. Am J Roentgenol.

[16] Kozłowski M., Piotrowska O., Giżewska-Kacprzak K. (2021). Omental infarction in a child—conservative management as an effective and safe strategy in diagnosis and treatment. IJERPH.

